# Primary cardiac angiosarcoma - a diagnostic roller-coaster till fatality

**DOI:** 10.34172/jcvtr.025.33285

**Published:** 2025-06-28

**Authors:** Bhupendra Kumar Sihag, Ajay Bahl, Sarthak Wadhera, Arnav Aggarwal, Mohsin Raj Mantoo, Atit A Gawalkar

**Affiliations:** ^1^Department of Cardiology, Post Graduate Institute of Medical Education and Research, Chandigarh, India; ^2^Department of Internal Medicine, Post Graduate Institute of Medical Education and Research, Chandigarh, India; ^3^Department of Cardiology, All India Institute of Medical Sciences, New Delhi, India

**Keywords:** Cardiac mass, Angiosarcoma, Cardiac imaging, Pericardial effusion

## Abstract

A 28-year-old male with a relatively short history of progressive dyspnea and a large pericardial effusion with tamponade was found to have an intracardiac mass localized in right atrium (RA) on echocardiography. Multimodality imaging revealed an irregular mass abutting the lateral wall of RA, with infiltration into surrounding pericardium and superior venacava. Positron emission tomography (PET) scan confirmed the mass as metabolically active lesion, along with uptake in mediastinal structures and lymph nodes. After an unrewarding percutaneous endomyocardial biopsy, open surgical biopsy was performed. Histologic examination confirmed the diagnosis of cardiac angiosarcoma. Unfortunately, patient had refractory shock and recurrent massive pericardial effusion (hemorrhagic) after biopsy and succumbed. The case highlights diagnostic dilemma of pericardial effusion in tuberculosis-endemic areas, role of multi-modality imaging in confirming cardiac malignancy and poor outcome of such patients.

## Introduction

 Primary malignant cardiac tumors are rare, the commonest subtype being angiosarcoma.^1^ It is an aggressive tumor that most commonly originates in the right atrium and spreads rapidly into other chambers, blood vessels, valves and pericardium. It also frequently metastasizes to distant organs like lungs, liver and lymph nodes.^1^ Prognosis of patients is very dismal, 1-year mortality in absence of surgical resection as high as 90% has been described.^2,3^

## Case Presentation

**Figure 1:**
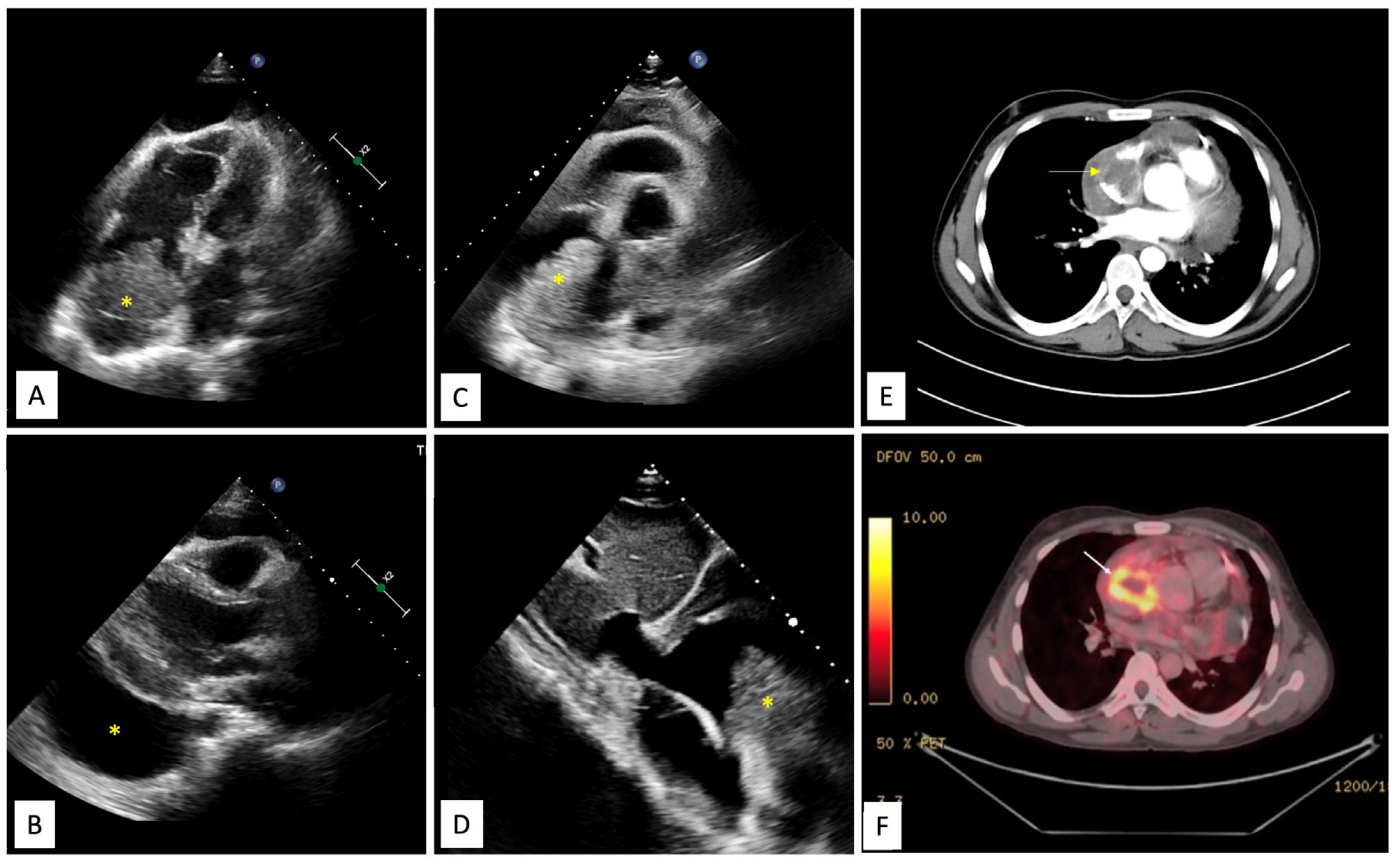
Echocardiography, CT and PET findings. A. Echocardiographic subcostal four chamber view demonstrating large multilobulated mass in the right atrium (yellow asterisk) with pericardial effusion. B. Echocardiographic parasternal long axis view showing large pericardial effusion (yellow asterisk). C. Echocardiographic parasternal short axis view showing large multilobulated mass in the right atrium close to the superior vena cava (yellow asterisk). D. Echocardiographic subcostal bi-caval view showing lobulated mass in the right atrium (yellow asterisk). E. A contrast enhanced computed tomographic image shows an irregular, broad-based, ‘cauliflower like’ mass abutting the lateral wall of the right atrium, with local infiltration into pericardium and a moderate pericardial effusion. F. Corresponding positron emission tomography (PET) image depicts high fluorodeoxyglucose (FGD) uptake by the right atrial mass, suggesting a metabolically active tumor, likely malignancy.

 A 28-year-old male presented with a history of progressive shortness of breath and dry cough for the past 1 month. There was no history of chest pain, hemoptysis, loss of weight or appetite or any family history of premature cardiac disease. On evaluation at a local clinic, his chest radiograph showed an enlarged cardiac silhouette and a point-of-care ultrasound revealed a large pericardial effusion, prompting a referral to our centre. At presentation to our hospital, the patient had tachycardia, tachypnea, jugular venous distention, clear lungs, muffled heard sounds and pulsus paradoxus suggestive of pericardial tamponade. Echocardiography revealed a large circumferential pericardial effusion (maximum 20mm anterior to right ventricle) along with right ventricle diastolic collapse. We also observed a 4.9 × 4.3 cm mass attached to the roof of right atrium (RA) (Figure 1A, 1B, 1C and 1D). A pericardial pigtail catheter was placed for drainage of effusion, relieving patient’s tamponade. Differentials for RA mass include thrombus, myxoma, primary malignant neoplasm or a metastasis. Pericardial fluid analysis had a leukocyte count of 300 cells/mm^3^ (99% lymphocytes), glucose 17mg/dl, protein 6.6g/dl and adenosine deaminase level 50.9U/L. Cytology (repeated thrice) revealed a predominantly mononuclear cell infiltrate with no atypical/malignant cells. Gram stain, bacterial culture, acid-fast stain & MGIT (Mycobacterial growth indicator tube) culture were negative. Considering the high prevalence of tuberculosis in the region, lymphocyte-predominant effusion and lack of malignant cells in the fluid, he was started empirically on anti-tubercular therapy (ATT).

**Figure 2:**
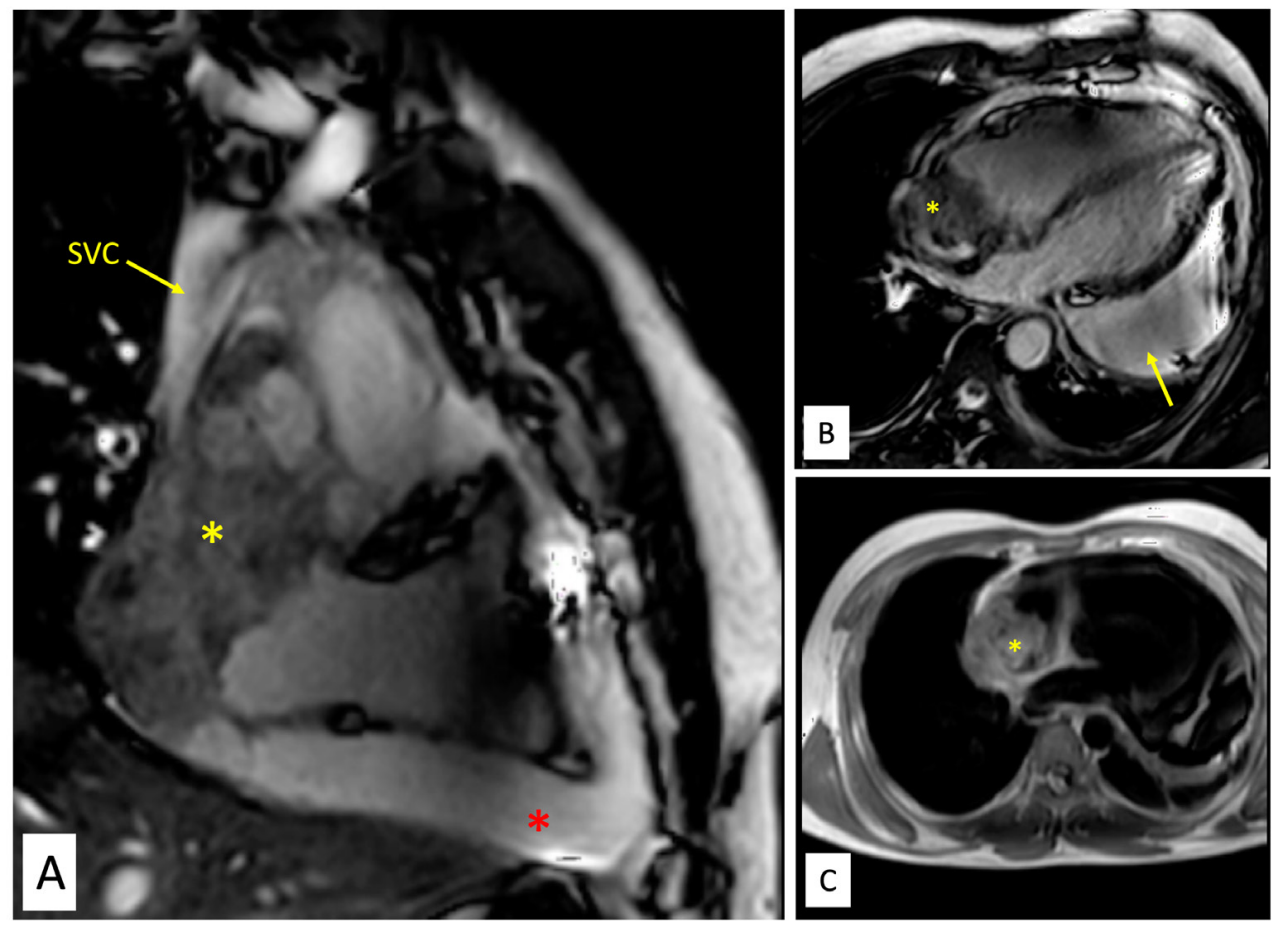
Magnetic resonance imaging of the tumour. A. Balanced steady state free precession (SSFP) TRUFisp image in coronal view shows craniocaudal extent of the mass(yellow asterisk) spanning from superior vena cava (SVC)-right atrial (RA) junction cranially to acute margin of the heart. The mass is seen infiltrating into SVC (yellow arrow). Large pericardial effusion is also seen (red asterisk). B. Balanced steady state free precession (SSFP) TRUFisp image in horizontal long axis shows heterogeneous hyperintense lobulated mass (yellow asterisk) extending into the right atria and superior vena caval junction. The mass is abutting the anterior wall of RA and interatrial septum. Additionally, asymmetric loculated moderate pericardial effusion is noted (yellow arrow). C. Axial T1 non-fat saturated image reveals heterogeneous signal intensity infiltrative mass (yellow asterisk) centered along anterior right atrial wall involving pericardium and extrapericardial space.

 Contrast enhanced cardiac computed tomography (CT) showed an irregular, broad-based, ‘cauliflower like’ mass abutting the lateral wall of RA with a heterogeneous streaky (sun ray) post-contrast enhancement (Figure 1E). There was local pericardial infiltration with associated moderate pericardial effusion. An aggressive mass in RA, metastasis or angiosarcoma, was suspected. There was no evidence of any primary tumor anywhere in chest or abdomen on CT scan. Cardiac magnetic resonance (CMR) imaging showed a T2-hyperintense mass measuring 64 mm x 44mm involving anterolateral wall of RA, with thickening of adjacent pericardium, superiorly invading cavo-atrial junction and extending into the superior venacava (SVC) (Figure 2A-2C). Invasion of SVC favours angiosarcoma over lymphoma; lymphoma usually presents as an insinuating mass without displacement or compression of vascular structures. The rapidly growing mass in the CMR compared to the CT image 1 week earlier, infiltration into pericardium and SVC pointed towards a rapidly growing malignant tumor. ATT was discontinued as there was a lack of improvement in symptoms and imaging evidence of likely malignancy. Positron emission tomography (PET) scan revealed an FDG-avid mass in the RA wall with extension into mediastinal structures and similar uptake in right lower paratracheal, prevascular, paraortic & paracardiac regions (Figure 1F). The mass was seen to infiltrate ascending aorta, pulmonary artery and SVC. It showed areas of photopenia & hypodensity within mass, suggestive of necrosis. No metastasis to distant organs was noted. Patient had further deterioration of dyspnea and in search of a definitive diagnosis, an endomyocardial biopsy was planned after discussion with heart team. The tissue analysis, however, did not yield any malignant cells. After discussion with the family, open biopsy using a lateral thoracotomy approach was performed. A few hours after the procedure, patient had sudden and rapidly progressive shock and worsening of respiratory distress requiring mechanical ventilation. Echocardiogram revealed large pericardial effusion with tamponade, which on aspiration yielded hemorrhagic fluid. With a possibility of active bleed from the vascular tumor or a vessel, a midline thoracotomy was done to localize and stop bleeding. The patient’s hemodynamics worsened during the procedure and he succumbed. Histological examination of the biopsied tissue revealed large areas of hemorrhage with fibrin & necrosis. Preserved areas showed a tumour arranged in sheets and short intersecting fascicles. There were slit like spaces with red blood cells. Tumor cells were moderately pleomorphic, had round-to-oval nuclei with vesicular chromatin, conspicuous nucleoli and moderate amount of cytoplasm. These cells were positive for CD31(membranous) and negative for desmin and PanCK(pancytokeratins) which was suggestive of angiosarcoma.

## Discussion

 The differential diagnoses for cardiac masses include thrombus, vegetation or tumor. The initial suspicion is often guided by the epidemiology, nature, onset and progression of symptoms, constitutional features and examination findings. Imaging findings like location, size, shape, site of attachment, mobility, contents and other characteristic features further help in delineating the true nature of the mass. Echocardiography is the primary modality for the diagnosis of intracardiac mass. Further, higher resolution multimodality imaging enables detailed characterization and facilitates operative planning.

 Angiosarcoma appears as a heterogeneously enhancing mass on CT scan with areas of necrosis and hemorrhage, along with pericardial effusion and infiltration into surrounding structures. Lymphoma appears as single or multiple isoattenuating mass relative to myocardium, lack of tumour necrosis and infrequent valvular involvement.^4-6^ CMR showing features like heterogeneity of signal intensity, local infiltration and gadolinium enhancement points toward malignancy. Angiosarcoma usually present as large infiltrative mass with heterogeneous signal intensity, isointense on T1, hyperintense on T2 with intratumoral hemorrhage and areas of signal void reflecting blood flow within vascular channels. Avid first pass and late gadolinium enhancement along vascular channels gives a characteristic “sunray” appearance. Lymphomas, on other hand, usually appear as multiple solid myocardial-based masses, which are isointense on T1, mildly hyperintense on T2, display heterogeneous LGE and do not show necrosis.^4,5^ An SUV max cut off value of 3.44 (sensitivity of 100% and specificity of 92.9%) and TVRmax of 1.55 (sensitivity 95.8% and specificity of 92.9%) in FDG-PET can identify malignant nature of a cardiac mass.^7^

 In cases of suspected cardiac angiosarcoma, pericardial fluid analysis, while helpful, often has a low diagnostic yield, with cytology frequently being negative, and a definitive diagnosis often requiring tissue biopsy.^8^ In the index case, imaging modalities could establish the malignant nature of the mass but could not adequately help in definite diagnosis. Such situations warrant consideration of minimally invasive (percutaneous needle aspiration or endomyocardial biopsy) or surgical approach (thoracotomy and open biopsy). The infiltrating nature of the intracardiac mass into the myopericardial plane along with rapidly increasing size raised the suspicion of malignant tumor. Primary tumors of the heart are rare.^9^ Most of the primary cardiac tumors are benign and among the malignant ones most are sarcomas(75%) followed by lymphoma and mesothelioma.^9^ The median survival of patients with cardiac sarcomas in a larger series is 6 months, which tends to improve with the complete surgical resection in earlier stages.^10^

 Angiosarcoma, the most aggressive form, accounts for around one third of the primary cardiac sarcomas.^11^ With a predilection for young males (3^rd^ to 5^th^ decade of life), it most commonly involves right atrium. It is characterised by rapid infiltration of surrounding cardiac and vascular structures with friability and tendency to bleed.^2^ It can present clinically with constitutional symptoms, pericardial effusion, symptoms of right heart failure by obstructing the venous return to the right side, supraventricular arrhythmias secondary to infiltration or symptoms related to distant metastasis such as hemoptysis (lung is the commonest site of metastasis).^1^ Wide surgical resection and relieving the mechanical consequence is primary modality of treatment especially when the disease is localised where it can be potentially curative or be a part of palliation with median survival of 14 months.^2,3^ Among patients who are managed with medical therapy alone, 90% die within 9–12 months.^3^ A multidisciplinary approach of radical surgical resection, systemic chemotherapy and radiation therapy is seen to provide the best chance of survival of up to 3 years.^12^ A combination of resection, chemotherapy, and radiation therapy has led to reports of survival up to 3 years.^12,13^ Cisplatin, cyclophosphamide, dacarbazine, doxorubicin, ifosfamide, mitomycin-C, paclitaxel, and vincristine are commonly prescribed agents in standard chemotherapy while immunotherapy has also been tried.^2,14^

## Conclusion

 Angiosarcoma is a highly aggressive cardiac tumor. Invasion of surrounding pericardium, other cardiac chamber and vessels suggest a malignant nature of cardiac mass. The prognosis is dismal, with early surgical resection being the treatment of choice in suitable patients.

## Competing Interests

 The authors declare that they have no competing interests.
